# Soil microarthropods alter the outcome of plant-soil feedback experiments

**DOI:** 10.1038/s41598-018-30340-w

**Published:** 2018-08-09

**Authors:** Eliška Kuťáková, Simone Cesarz, Zuzana Münzbergová, Nico Eisenhauer

**Affiliations:** 1 Department of Botany, Faculty of Science, Charles University in Prague, Benátská 2, 128 01 Praha 2, Czech Republic; 2 Institute of Botany, Czech Academy of Sciences, v. v. i., Zámek 1, 252 43 Průhonice, Czech Republic; 3 German Centre for Integrative Biodiversity Research (iDiv) Halle-Jena-Leipzig, Deutscher Platz 5e, 04103 Leipzig, Germany; 4 Institute of Biology, Leipzig University, Deutscher Platz 5e, 04103 Leipzig, Germany; 5 Friedrich Schiller University of Jena, Institute of Ecology, Dornburger Str. 159, 07743 Jena, Germany

## Abstract

Plant-soil feedback (PSF) effects are studied as plant growth responses to soil previously conditioned by another plant. These studies usually exclude effects of soil fauna, such as nematodes, soil arthropods, and earthworms, although these organisms are known to influence plant performance. Here, we aimed to explore effects of a model microarthropod community on PSFs. We performed a PSF experiment in microcosms with two plant species, *Phleum pratense* and *Poa pratensis*. We added a model microarthropod community consisting of three fungivorous springtail species (*Proisotoma minuta*, *Folsomia candida*, and *Sinella curviseta*) and a predatory mite (*Hypoaspis aculeifer*) to half of the microcosms. We measured seedling establishment and plant biomass, nematode and microbial community composition, microbial biomass, and mycorrhizal colonization of roots. Microarthropods caused changes in the composition of nematode and microbial communities. Their effect was particularly strong in *Phleum* plants where they altered the composition of bacterial communities. Microarthropods also generally influenced plant performance, and their effects depended on previous soil conditioning and the identity of plant species. Microarthropods did not affect soil microbial biomass and mycorrhizal colonization of roots. We conclude that the role of soil microarthropods should be considered in future PSF experiments, especially as their effects are plant species-specific.

## Introduction

The composition and dynamics of plant communities strongly depend on plant-soil interactions^1^. To better understand the role of plant-soil interactions, plant-soil feedback experiments have been widely applied^2^. The concept of plant-soil feedback effects is based on the idea that plants change properties of the soil in which they grow, and such modified soil can, in turn, influence their growth^3^. Specifically, plants can induce changes in soil biotic (e.g., composition of microbial communities) and abiotic (e.g., nutrient content or pH) properties^4^. Several studies have regarded plant-soil feedbacks as one of the key mechanisms affecting species coexistence in natural communities, the course of vegetation succession, and spread of invasive species^2^. However, with such a wide applicability in various areas of plant and soil ecology, there is a need for precise interpretation of experimentally measured plant-soil feedback effects^5^.

Plant-soil feedbacks are often studied using two-phase experiments. The first phase of such experiments is a conditioning phase, when a plant is grown in a pot to modify soil properties. The second phase is a feedback phase, when another plant (of the same or different species) is grown in the soil modified during the conditioning phase. The final biomass of a plant from the feedback phase is compared to the biomass of a plant grown in a control soil. Because soil microorganisms are perceived as the main drivers of plant-soil feedback effects^6,7^, many studies focus solely on the effects of the microbial community by transferring microbial inoculum between the two experimental phases. This excludes not only the abiotic compound of feedback effects from the experiment, but also major groups of soil fauna, such as microarthropods. Other plant-soil feedback studies use whole soil^8,9^, which should lead to results which are more comparable to natural conditions. However, even this approach can exclude or at least reduce densities of soil fauna: unless these organisms are added to the experiment on purpose or their natural immigration is allowed during the experiment. Thus, to contain larger soil fauna like mesofauna, the soil should be obtained from a natural locality, carefully transported and treated. Treatments like drying or freezing can be lethal for soil mesofauna, but also soil sieving can lead to substantially decreased densities if they are larger than the used mesh size or sensitive to disturbances caused by processing the soil^10^. As a result, most plant-soil feedback experiments may have unintentionally excluded or at least under-estimated the effect of soil mesofauna.

The suppression of soil mesofauna in plant-soil feedback experiments can be expected to significantly influence the respective results. Soil mesofauna is a significant component of the plant-soil system^11,12^. Beside the direct effect of root-feeders on plants, some soil mesofauna can influence interactions between plants and microbial communities by feeding on specific microorganisms. For example, springtails are known to feed on fungal hyphae^13^, and thus indirectly influence plant growth by altering mycorrhizal symbiosis^14–17^. Springtails’ grazing on mycorrhizal fungi can support growth of soil bacteria by lowering fungal biomass and thus making more resources accessible to bacterial communities^17^. In this respect, springtails can indirectly impact plant-microbe competition for nutrients^18^. To sum up, soil microarthropods act on many stages of plant-soil interactions, having impact on both soil biota and nutrient cycling^19,20^. In previous studies, it has been shown that inclusion of larger soil organisms in experiments can change nutrient allocation patterns in plants^17,21^, plant growth^21,22^, and alter plant community composition^23–25^. However, the role of soil fauna in plant-soil feedback effects is still unclear.

In this study, we investigated the effect of a model soil microarthropod community on plant-soil feedback effects. We set up a common plant-soil feedback experiment using two grassland plant species that are known to experience dissimilar plant-soil feedback effects^26^: *Phleum pratense* (in the following: *Phleum* for brevity) known for its positive intraspecific feedback effect, and *Poa pratensis* (in the following: *Poa* for brevity) that exerts a negative intraspecific feedback effect, both of them probably caused by interactions with plant species-specific soil microbial communities^26^. By following common methodology in plant-soil feedback studies as mentioned above, we established a two-phase experiment consisting of a soil conditioning phase and a feedback phase. Furthermore, we used a model microarthropod community comprising two trophic levels: three springtail species as primary consumers (fungivores), and a predatory mite as a predator of springtails and other small invertebrates occurring in the soil. We hypothesized that (1) the addition of soil microarthropods will change the plant response to the pre-conditioned soil, and that (2) the effect of microarthropods will be context-dependent, i.e., it will be plant species-specific and depend on soil conditioning, as microarthropods will influence the composition of species-specific soil microbial communities responsible for the feedback effect.

## Results

### Effects on plants

**Figure 1:**
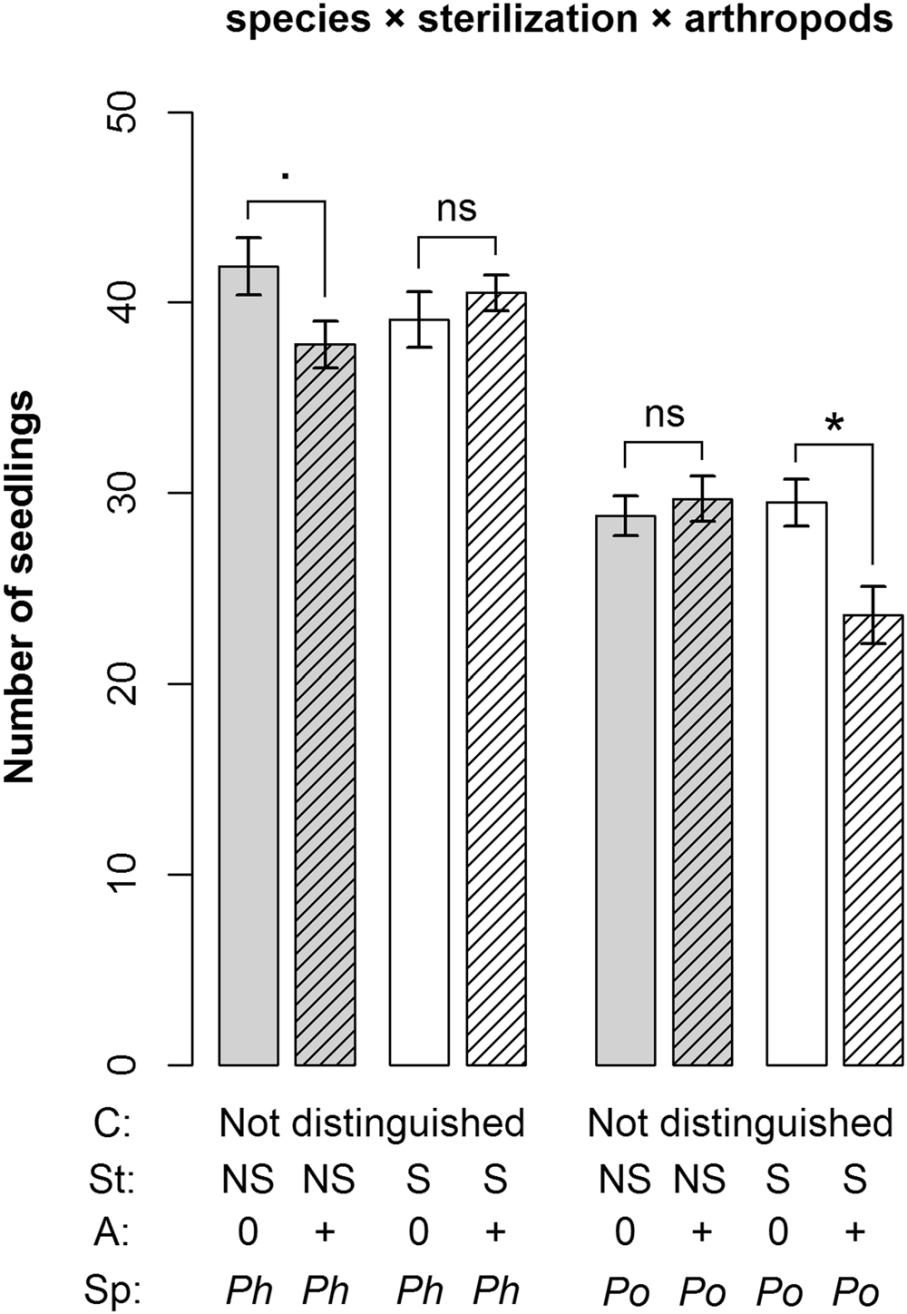
Number of established seedlings as influenced by the interaction of sterilization, arthropod addition and species grown in the feedback phase. Shown are means ± SE. Symbols above bars indicate significant differences between treatments with and without arthropods addition (*p < 0.05; ^.^p < 0.1; ns p > 0.1). Grey bars represent non-sterilized soil, white bars sterilized soil; hatched bars represent treatments with arthropod addition. C = conditioning species, *Ph* = *Phleum*, *Po* = *Poa*; St = sterilization, NS = non-sterilized, S = sterilized; A = arthropod treatment, 0 = no arthropods added, +  = arthropods added; Sp = plant species in feedback phase; Not distinguished = this treatment is not distinguished in the respective analysis.

**Figure 2:**
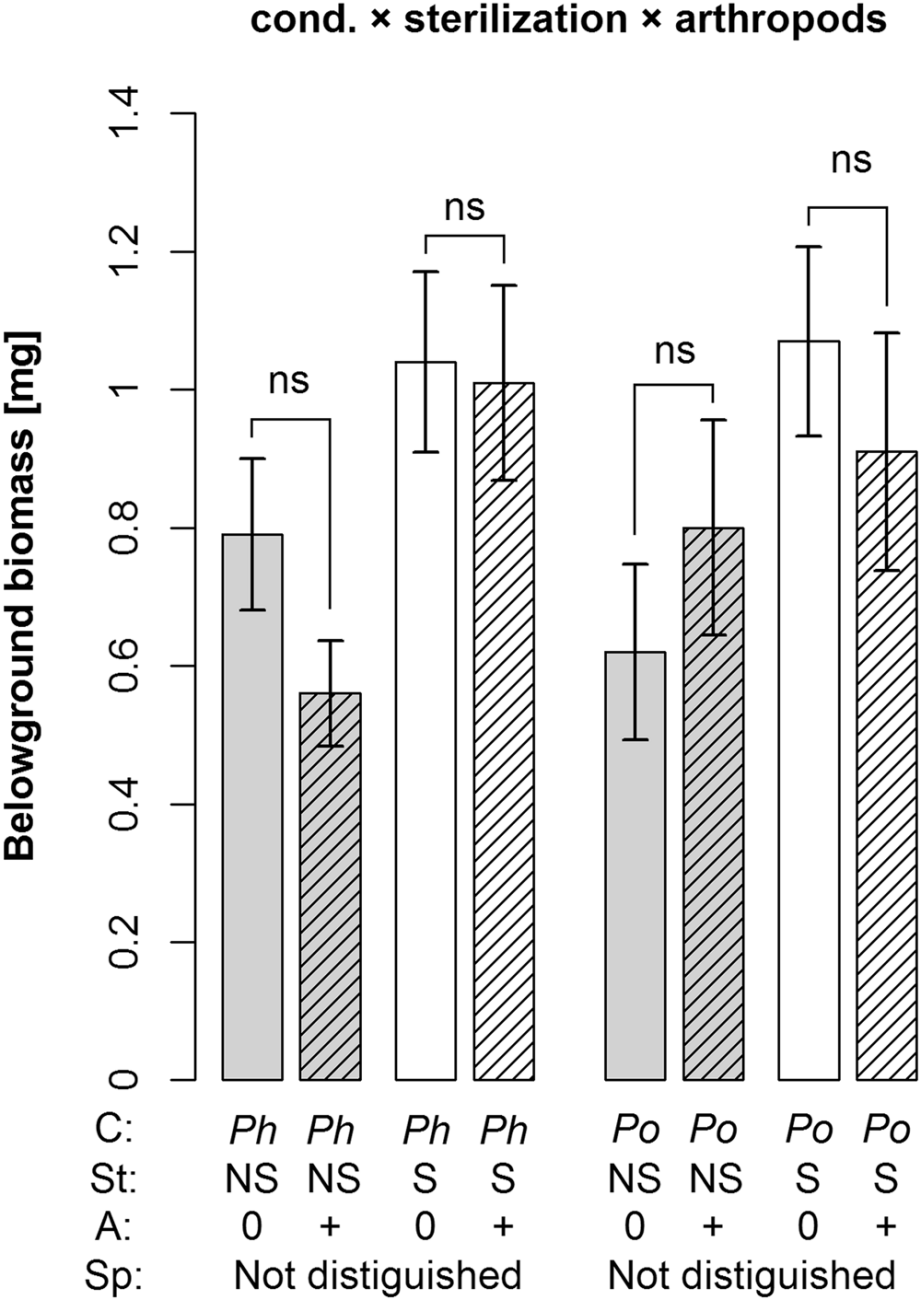
Belowground biomass as affected by the interaction of soil conditioning, sterilization and arthropod addition. Shown are means ± SE. Symbols above bars indicate significant differences between treatments with and without arthropods addition (*p < 0.05; ^.^p < 0.1; ns p > 0.1). If distinguished, grey bars represent non-sterilized soil, white bars sterilized soil, and hatched bars treatments with arthropod addition. C = conditioning species, *Ph* = *Phleum*, *Po* = *Poa*; St = sterilization, NS = non-sterilized, S = sterilized; A = arthropods, 0 = no arthropods added, + = arthropods added; Sp = plant species in feedback phase; Not distinguished = this treatment is not distinguished in the respective analysis.

**Figure 3:**
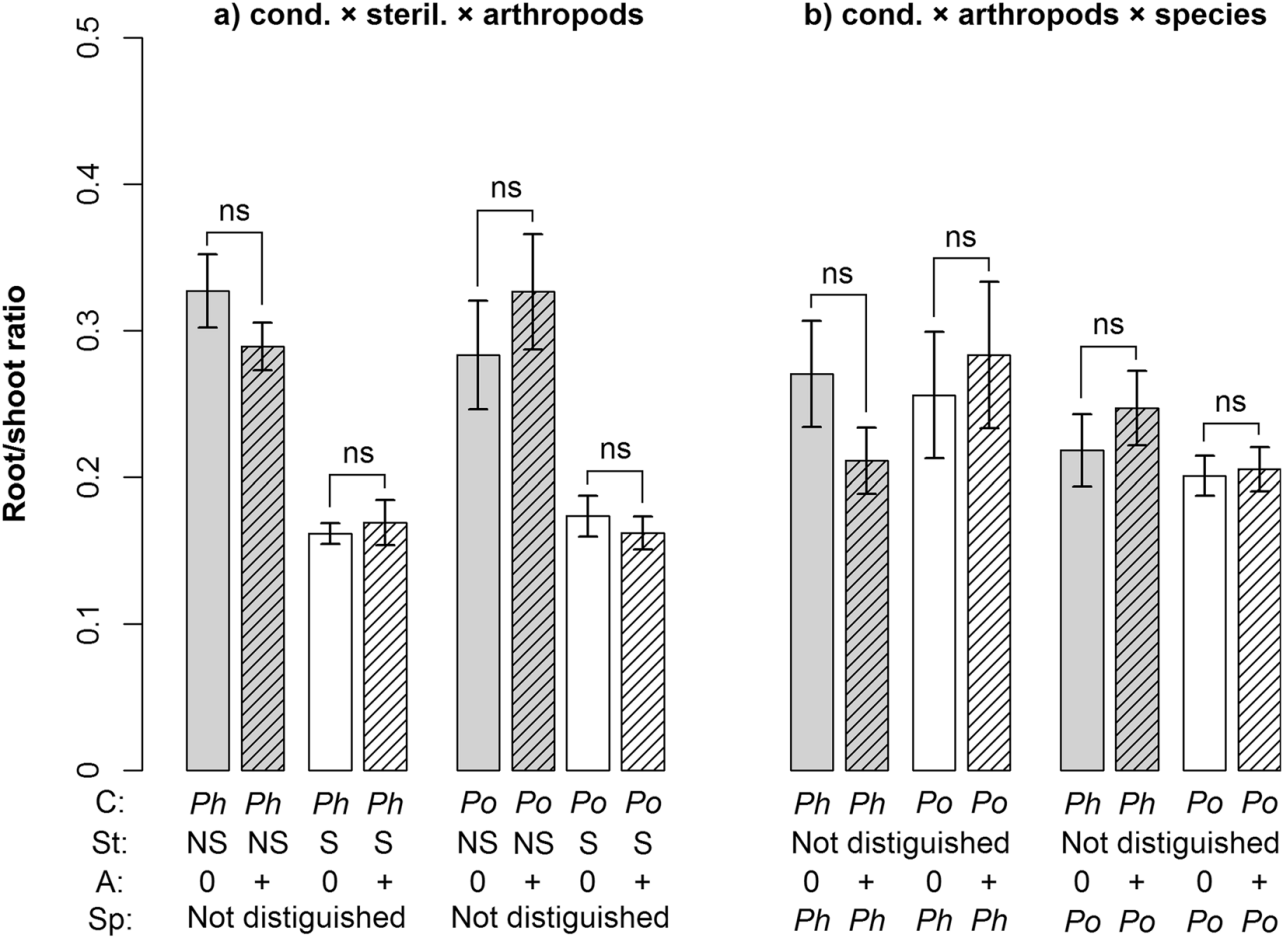
Root-to-shoot ratio as influenced by the interaction of **(a)** conditioning, sterilization and arthropod addition; **(b)** conditioning, arthropod addition and plant species grown in the feedback phase. Shown are means ± SE. Symbols above bars indicate significant differences between treatments with and without arthropods addition (*p < 0.05; ^.^p < 0.1; ns p > 0.1). Grey bars represent non-sterilized soil, white bars sterilized soil, and hatched bars treatments with arthropod addition. C = conditioning species, *Ph* = *Phleum*, *Po* = *Poa*; St = sterilization, NS = non-sterilized, S = sterilized; A = arthropods, 0 = no arthropods added, + = arthropods added; Sp = plant species in feedback phase; Not distinguished = this treatment is not distinguished in the respective analysis.

The two plant species differed both in seedling establishment and final biomass. *Phleum* had significantly higher seedling establishment and also produced more above- and belowground biomass than *Poa* (Figs 1–3, Table 1). Seedling establishment was influenced by the interaction of soil conditioning × sterilization: the sterilization treatment increased seedling establishment in *Phleum* soil, but decreased it in *Poa* soil (Fig. S1). Both species produced more above- and belowground biomass in sterilized soils (Fig. 2 and S2, Table 1). Aboveground biomass was influenced by the three-way interaction of soil conditioning × sterilization × species: both plant species grew better in soil conditioned by the other species, and sterilization increased the differences between this intra- and interspecific soil feedback effect (Fig. S2, Table 1). Similarly, each species produced more belowground biomass in soil conditioned by the other species (Fig. S3) but there was no interaction with the sterilization treatment (Table 1). The root-to-shoot ratio was significantly higher in sterilized soils for both species, but the ratio for *Phleum* was generally higher across the other treatments (Fig. 3, Table 1). We found a significant effect of the interaction of soil conditioning × sterilization × species on the root-to-shoot ratio (Table 1): the ratio in both plant species was the highest when they were grown in non-sterilized soil conditioned by the other species (Fig. S4). The root-to-shoot ratio in sterilized soils was much lower, especially in the case of *Phleum* plants (Fig. S4).

**Table 1:** The effect of treatments and their interactions on seedling establishment, plant aboveground and belowground biomass, root/shoot ratio, and soil microbial biomass.

	Germination		Biomass				Root/shoot ratio		Microbial biomass	
			Aboveground		Belowground					
	F	p	F	p	F	p	F	p	F	p
Sterilization	2.20	0.143	**178**.**57**	**<0**.**001**	**15**.**77**	**<0**.**001**	**91**.**02**	**<0**.**001**	**15**.**06**	**<0**.**001**
Species	**165**.**72**	**<0**.**001**	**62**.**56**	**<0**.**001**	**45**.**09**	**<0**.**001**	**6**.**58**	**0**.**013**	**13**.**63**	**<0**.**001**
Conditioning	0.79	0.376	0.03	0.855	0.00	0.962	0.01	0.919	0.43	0.517
Arthropods	**4**.**32**	**0**.**042**	0.82	0.367	0.61	0.437	0.01	0.919	0.63	0.432
Steril. × species	2.05	0.157	**13**.**33**	**0**.**001**	0.79	0.377	**14**.**67**	**<0**.**001**	**30**.**40**	**<0**.**001**
Steril. × cond.	**5**.**02**	**0**.**029**	0.03	0.867	0.20	0.659	0.04	0.838	***3***.***95***	***0***.***051***
Species × cond.	1.11	0.297	**4**.**62**	**0**.**035**	***3***.***92***	***0***.***052***	***3***.***93***	***0***.***052***	0.59	0.445
Steril. × arthropods	0.12	0.727	0.49	0.486	0.20	0.654	0.02	0.892	0.03	0.857
Species × arthropods	0.39	0.537	0.05	0.828	0.08	0.777	1.20	0.278	2.48	0.120
Cond. × arthropods	0.21	0.648	0.01	0.907	0.84	0.363	1.20	0.278	1.01	0.318
Steril. × species × cond.	2.20	0.143	**4**.**17**	**0**.**045**	0.02	0.896	**7**.**30**	**0**.**009**	1.04	0.312
Steril. × species × arthropods	**11**.**02**	**0**.**001**	1.17	0.283	1.05	0.310	0.14	0.708	1.20	0.278
Steril. × cond. × arthropods	0.39	0.537	1.02	0.317	***2***.***98***	***0***.***089***	***2***.***81***	***0***.***099***	0.81	0.373
Species × cond. × arthropods	0.89	0.348	0.25	0.616	2.07	0.155	***3***.***80***	***0***.***056***	0.41	0.522
Steril. × species × cond. × arth.	0.70	0.406	1.02	0.315	0.04	0.841	1.69	0.198	0.48	0.490

The results of ANOVA are shown. Bold values indicate significant relationships (p < 0.05), values in italics and bold indicate marginal significance (p < 0.1).

The presence of arthropods influenced seedling establishment in interaction with sterilization and species (sterilization × arthropod addition × species interaction; Table 1). While seedling establishment of *Phleum* in sterilized soil and that of *Poa* in non-sterilized soil was not influenced by arthropods, their addition decreased seedling establishment of *Phleum* in non-sterilized soils and that of *Poa* in sterilized soils (Fig. 1). We also found a marginally significant interaction effect of soil conditioning × sterilization × arthropods on belowground biomass (Table 1). Arthropod addition generally decreased belowground biomass of plants grown in *Phleum* soil, although the decrease in sterilized *Phleum* soil was much weaker than in non-sterilized soil. In sterilized *Poa* soil, arthropod addition also decreased belowground biomass, but, in contrast, it increased belowground biomass in non-sterilized *Poa* soil (Fig. 2). The arthropod addition treatment also altered the root-to-shoot ratio in interaction with soil conditioning and plant species, or soil conditioning and sterilization (Fig. 3, Table 1). Specifically, arthropods lowered the root-to-shoot ratio of plants in non-sterilized *Phleum* soil, but they increased it in non-sterilized *Poa* soil. In sterilized soils, the changes were much weaker (Fig. 3a). In *Phleum* soils, arthropod addition lowered the root-to-shoot ratio of *Phleum* plants, but increased the ratio of *Poa* plants (Fig. 3b).

### Effects on soil microbial and nematode communities

All of the measured soil biological properties were affected by the sterilization treatment: sterilization caused an increase in soil microbial biomass (+8.3%; Table 1; Fig. S5), but largely decreased both nematode abundance (−94.1%; *χ*^2^ = 27.65; p < 0.001) and mycorrhizal colonization of *Phleum* roots (−99.9%; decrease to 0.03 ± 0.02% roots colonized; *χ*^2^ = 36.10; p < 0.001;). Soil microbial biomass was significantly influenced by the interaction of soil conditioning × sterilization (Table 1): it increased by the sterilization treatment in *Phleum*-conditioned soil, whereas there was no effect of sterilization in *Poa* soil (Fig. S5a). Similarly, microbial biomass associated with *Phleum* plants was higher in sterilized soil, while for *Poa* plants, the pattern was the opposite (Fig. S4b). In non-sterilized soil, the nematode abundance was influenced by soil conditioning treatment (7.4 ± 2.8 nematodes per g soil dry weight in *Phleum* conditioned soil compared to 5.6 ± 1.7 in *Poa* soil; F = 4.92; p = 0.03), but not by the plant species, arthropod addition, or their interactions (p ≥ 0.26).

**Figure 4:**
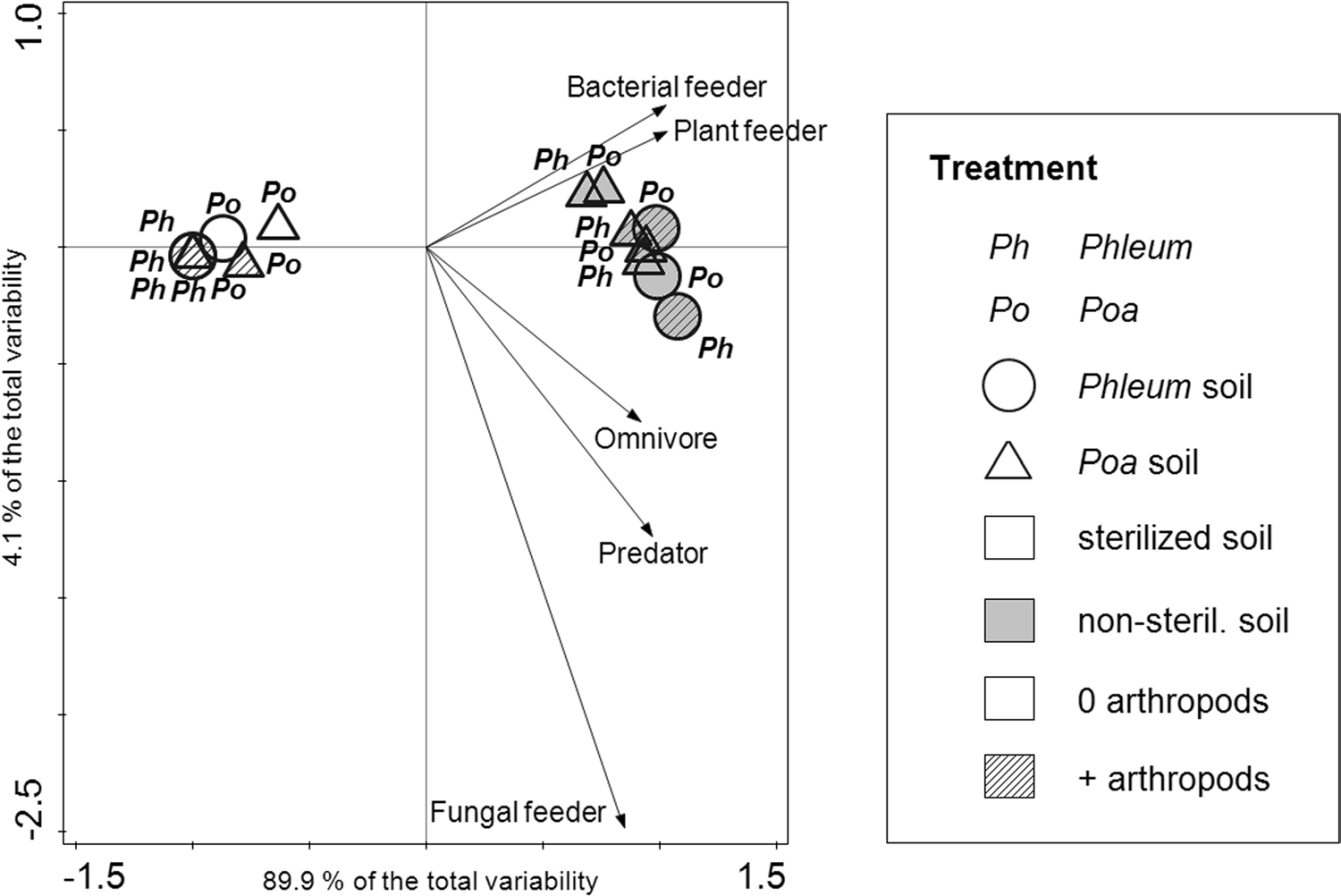
PCA of the composition of nematode trophic groups after the feedback phase. Symbols represent the individual experimental treatments (centroids). Arrows indicate the individual nematode trophic groups.

**Figure 5:**
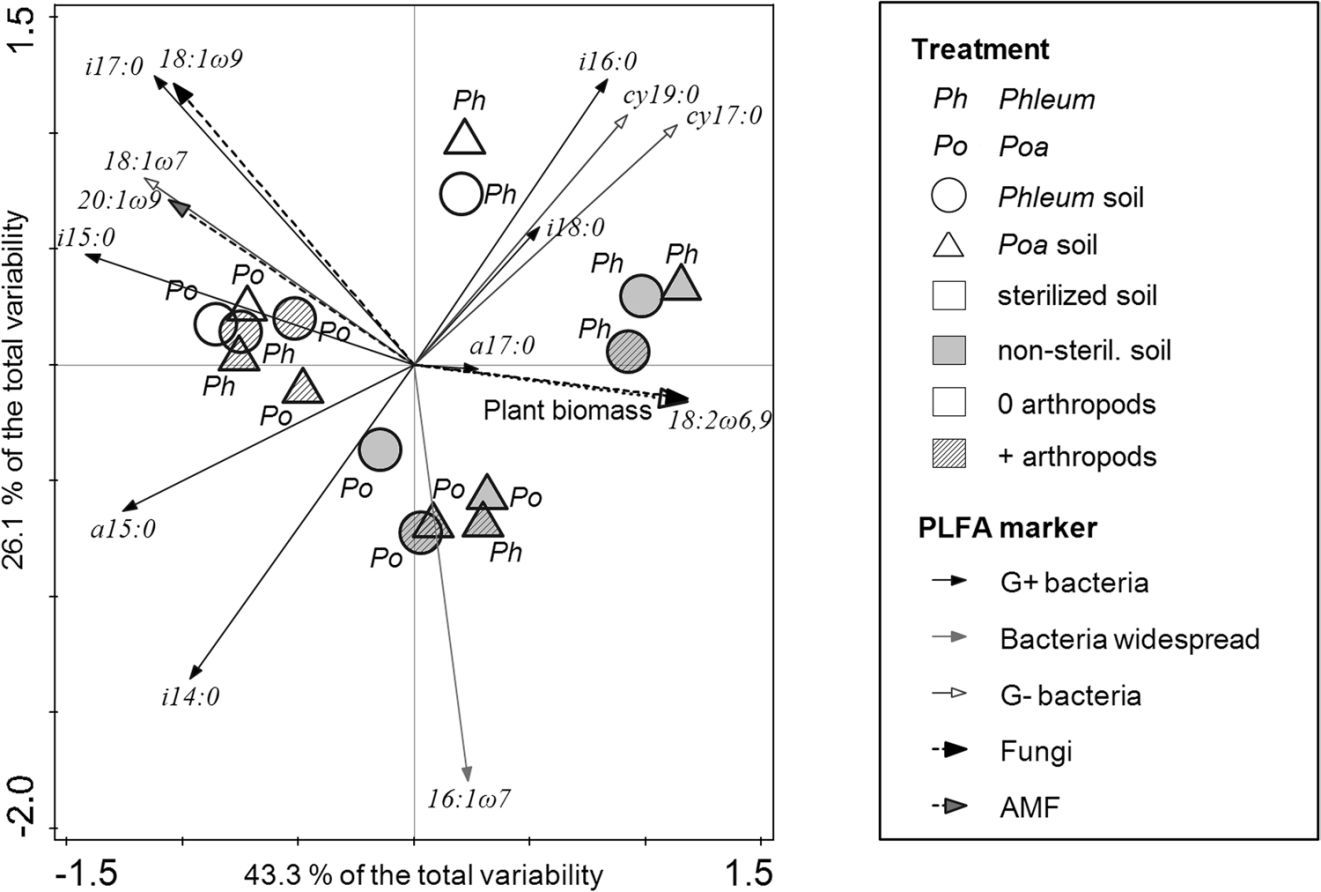
PCA of the composition of microbial communities after the feedback phase. Symbols represent individual experimental treatments (centroids of samples). Arrows indicate the individual PLFA markers.

The functional composition of the nematode community was marginally significantly influenced by the four-way interaction of all experimental treatments, with the strongest direct effect of sterilization (Table 2, Fig. 4). In non-sterilized soil, the greatest differences were observed between the two soil conditioning treatments with omnivores, predators, and fungal feeding nematodes found preferentially in *Phleum*-conditioned soil (Fig. 4). There was no direct effect of the species grown in the feedback phase on nematode community composition. The composition of soil PLFAs also responded significantly to the four-way interaction of all experimental treatments (Table 2). The highest differences in PLFA composition were observable between the sterilized and non-sterilized soil and between the two plant species grown in the feedback phase (Table 2, Fig. 5). However, there was no direct effect of the soil conditioning treatment. Sterilization increased the arbuscular mycorrhizal marker 20:1ω9, the fungal marker 18:1ω9, and the gram-positive markers i15:0 and i17:0. The microbial communities in sterilized soils were clearly separated by the first axis from communities in non-sterilized soils. The soils containing *Phleum* plants were associated with gram-negative markers cy17:0 and cy19:0 increased, as well as i16:0 as an indicator of gram-positive bacteria. The arthropod addition treatment increased the amount of PLFA markers i14:0 (gram-positive bacteria) and 16:1ω7 (widespread bacteria). Interestingly, conditioning of the soil with *Phleum* or *Poa* did not strongly influence PLFAs; sterilization and plant species grown in the feedback phase were of higher importance.

**Table 2:** The effect of the experimental treatments and their interactions on nematode and microbial (PLFA) community composition. Results from RDA are shown.

	Nematode community			PLFA composition		
	F	p	var. exp.	F	p	var. exp.
Sterilization	**389**	**<0**.**002**	**83**.**6%**	**56**.**00**	**<0**.**002**	**55**.**5%**
Species	1.9	0.13	1.2%	**18**.**30**	**<0**.**002**	**28**.**3%**
Conditioning	**3**.**0**	**0**.**028**	**2**.**6%**	1.10	0.314	0.2%
Arthropods	***2***.***1***	***0***.***084***	***1***.***4%***	**6**.**40**	**0**.**004**	**10**.**9%**
Steril. × species	***2***.***1***	***0***.***094***	***1***.***4%***	**7**.**60**	**<0**.**002**	**13**.**2%**
Steril. × cond.	2.5	0.052	2.0%	1.70	0.128	1.5%
Species × cond.	0.3	0.9	0.4%	0.70	0.602	0.0%
Steril. × arthropods	2.1	0.1	1.5%	0.50	0.784	0.0%
Species × arthropods	1.0	0.412	0.0%	5.40	**<0**.**002**	9.3%
Cond. × arthropods	0.5	0.764	0.0%	1.60	0.136	1.3%
Steril. × species × cond.	0.5	0.698	0.0%	1.50	0.136	1.3%
Steril. × species × arthropods	1.1	0.338	0.2%	**3**.**10**	**0**.**012**	**5**.**0%**
Steril. × cond. × arthropods	1.4	0.214	0.6	0.80	0.516	0.0%
Species × cond. × arthropods	1.0	0.38	0.1%	0.90	0.452	0.0%
Steril. × species × cond. × arth.	***2***.***3***	***0***.***062***	***1***.***9%***	**2**.**30**	**0**.**040**	**3**.**8%**

Bold values indicate significant relationships (p < 0.05), values in italics and bold indicate marginal significance (p < 0.1).

## Discussion

In this study, we show that the presence of soil microarthropods in a plant-soil feedback experiment can substantially alter the composition of soil microbial and nematode communities. More importantly and potentially as a consequence of these shifts in the microbial and nematode communities, the presence of microarthropods altered plant seedling establishment, belowground biomass, and biomass allocation patterns. The effect of microarthropods was plant species-specific and depended on previous soil conditioning.

The two plant species we used in our experiment exhibited contrasting plant-soil feedback effects (responded differently to soil conditioning treatments) as they did in the previous study of Cortois *et al*.^26^. However, although we detected significant plant-soil feedback effects on the biomass of plants at the end of the experiment, the seedling establishment rate was not significantly influenced. Similar differences between plant-soil feedback effects on seedling establishment and plant biomass have been described before^27,28^, suggesting that in each of these life stages, plant performance may be influenced by different mechanisms. For instance, plant pathogens are often specialized to a certain life stage of a host plant^29^, and several studies suggested that such specialization can be found also in mycorrhiza^30,31^. As a result, soil biota showing a certain effect at one life stage of a plant species might have a different impact at another life stage of the same species.

The two plant species were also associated with distinct microbial and nematode communities. The fact that plants facilitate specific communities of soil organisms^32,33^ is considered as one of the main drivers of the plant-soil feedback effects^6,34^. In our study, most of the soil samples from *Phleum* plants were associated with increased PLFA markers cy17:0, cy19:0 (gram-positive bacteria). Gram-negative bacteria are often faster growing than gram-positive bacteria, and their increased abundance in soils reflects higher nutrient availability and, generally, environments favouring r-strategists^35^. *Phleum* plants could thus facilitate gram-positive bacteria by higher production of root exudates compared to *Poa* plants that were generally smaller. Apparently, the bacterial communities responded to the plant species very quickly: in samples taken after the feedback phase, we could not detect any effect of original soil conditioning, but the effect of species grown in the feedback phase was very strong. By contract, nematode abundance and community composition of trophic groups at the end of the experiment were still affected by the plant species that conditioned the soil (conditioning phase). This difference in stability of these soil communities could be related to the different generation times of the respective organisms: while bacteria or fungi can produce multiple generations per day^36^, nematodes are slower, with generation turnover ranging from several days to months^37^. The composition of nematode communities could also be stabilized by other interactions with soil organisms, such as top-down regulation by the predatory mites. These differences in the stability of soil communities and, therefore different importance for plant-soil feedbacks, should be studied in future experiments.

Addition of the model microarthropod community in our experiment altered the composition of both nematode and microbial communities in the soil. The shifts in the soil communities were not systematic across all the treatments, but rather depended on interactions with the other experimental treatments. For example, microarthropods caused large shifts in PLFA composition in soils from *Phleum* plants, but not in soils from *Poa* plants. Microarthropods are generally known to influence the abundance and composition of microbial communities^17,38^. The fact that their influence can vary, depending on plant species or soil conditioning, is an important finding for plant-soil feedback research. Interestingly, although the springtails we used in the present study are considered to be mostly fungivorous, they did not affect the mycorrhizal colonization of *Phleum* roots. However, previous studies showed both increases and decreases in arbuscular mycorrhiza colonization rates in the presence of springtails, depending on springtail densities^39^ or the availability of alternative resources^16^, suggesting that the impact of springtails are context-dependent. In contrast, the addition of microarthropods largely influenced composition of bacterial PLFAs, probably indirectly, via changes in resources available to bacteria due to springtails’ grazing on soil fungi and plant roots^17^. The changes in microbial communities caused by microarthropods were reflected in their community composition rather than in the total biomass, since we did not detect differences in soil microbial biomass.

Microarthropod addition treatment not only affected soil microbial communities, but also had impact on plant performance in the feedback phase. Specifically, microarthropods altered seedling establishment, belowground plant biomass, and plant biomass allocation, with the effects depending on interactions with other treatments. Although it has been shown that springtails can feed on plant roots^40^, our results suggest that herbivory was not the only determinant of plant performance in our experiment. Rather, it is very likely that microarthropods altered plant growth also indirectly by feeding on soil microbial or nematode communities, since we reported shifts in both microbial and nematode communities caused by microarthropod addition treatment. Springtails can, for example, influence plant germination by feeding on the fungal coat of the seeds^41–43^. Their negative influence on seedling establishment in the present study could thus be caused by springtails’ feeding on mycorrhizal fungi^13^. Feeding on the germinating seeds themselves is unlikely as this behaviour of springtails is considered very rare^43^. Besides these effects, microarthropods can also alter the nutrient availability in the soil^19,20^. As plants are known to change their biomass allocation patterns according to the nutrient status of the soil^44,45^ and even according to the form of the nutrient source^46^, it is possible that the microarthropods impacted plant root-to-shoot ratio by altering soil nutrient availability.

We conclude that soil microarthropods can significantly alter plant-soil feedback effects. In our experiment, their presence altered the composition of soil microbial and nematode communities (i.e., the potential agents of plant-soil feedbacks) and plant performance. More importantly, their effect was highly specific as it differed between soil conditionings and was affected by prior soil sterilization. This illustrates that microarthropods do not impact plant-soil feedbacks systematically, but their effects can be context-dependent. Considering that soil microarthropods are significant members of soil biota, we propose that their role should be considered in future plant-soil feedback research.

## Methods

### Model species

We used two model plant species: *Poa pratensis* (*Poa* hereafter for brevity) and *Phleum pratense* (*Phleum* hereafter). The choice of these species was based on previous research in the framework of the Jena Experiment^26^, where they showed opposing plant-soil feedback effects. This study found that *Poa* exhibited negative intraspecific plant-soil feedback effects (i.e., it grew worse in soil conditioned by individuals of *Poa* than in soil conditioned by individuals of other species). In contrast, *Phleum* showed a positive intraspecific plant-soil feedback effect (i.e., it grew better in soil conditioned by individuals of the same species than in soil of other species). Such opposing feedbacks could be driven by different mechanisms and thus could interact differently with added microarthropods. Seeds of both species were obtained from the same commercial provider (Rieger-Hoffmann GmbH, Blaufelden-Raboldshausen, Germany) as in Cortois *et al*.^26^.

As model soil microarthropods, we used one species of predatory mite (*Hypoaspis aculeifer*; Gamasida) and three springtail species (*Proisotoma minuta*, *Folsomia candida*, and *Sinella curviseta;* Collembola). The mite species is a generalist predator feeding on other soil microarthropods including springtails^47^. The springtail species feed mostly on soil fungi (mycorrhiza as well as saprotrophic fungi), but they are omnivorous and can feed on a variety of other resources reaching from soil microfauna (such as Protozoa, Nematoda, or Rotatoria) and microflora (such as bacteria, and algae), to plant leaf litter or even living roots^40,48^. In this study, we used a model community consisting of three springtail species to increase realism of the experiment: using a single species can lead to biased results due to specific feeding preferences of the model species^49^. Families of these three species (Isotomidae and Entomobryidae) can be found in the grassland where the experimental soil was obtained^50^. The springtail species were cultured on yeast in the laboratory. The mite species was obtained from a commercial provider (Schneckenprofi in Germany) and was also used in previous laboratory studies and shown to feed on the three springtail species^51^.

### Plant-soil feedback experiment

Following common methodology in plant-soil feedback experiments^52^, our experiment consisted of two phases: the conditioning phase and the feedback phase. To set up the conditioning phase, we used soil from a diverse semi-natural grassland adjacent to the Jena Experiment, Jena, Germany^53^. The soil has a pH value of 8.1, carbon concentration of 4.6%, nitrogen concentration of 0.3%, and a C-to-N ratio of 15.7; lime content of ~6%, a clay content of ~14%, a silt content of ~41%, and a sand content of ~45%. The two plant species were grown in monocultures in a climate chamber, each species in two 20 l mesocosms. The growth conditions were 12 hours of daylight, 25 °C, and 70% humidity. The duration of the conditioning phase was 3 months. After that, plant shoots were harvested, and the soil was sieved with a mesh (size 5 mm) to remove plant roots. The soil of both mesocosms of each plant species was homogenized and sampled for further analyses. The approach of mixing soils after the conditioning phase has been used in other studies^8,9^. It might be argued that such a treatment leads to artificially lowered variation in soil feedback effects among replicates. However, the main goal of this study was not to measure effect sizes but to investigate the possible influence of soil microarthropods on plant-soil feedback effects, legitimizing the present approach^54^. Then, the soil was split in half, and one half of each soil type was sterilized by autoclaving to remove the specific soil microorganisms developed during the conditioning phase (twice, each 20 min at 120 °C^55^).

The experiment for the feedback phase was set up in 300 ml microcosms (diameter: 7 cm, height: 10 cm) with a fine mesh at the bottom (50 µm) and a 10 cm plastic fence at the top of the round pots, both preventing soil fauna from escaping. Microcosms were filled with each of the pre-conditioned soil (further referred to as *Poa*-conditioned soil and *Phleum*-conditioned soil, respectively), both either sterilized or non-sterilized. Each of these soil types was crossed with further treatments: sowing of either *Phleum* or *Poa* seeds, and either an addition of soil arthropods or no addition of arthropods. With five replicates per treatment, the experiment resulted in 80 microcosms: 2 (soil conditioning) × 2 (soil sterilization) × 2 (species sown) × 2 (arthropod addition) × 5 (replicates).

Before sowing, all microcosms were repeatedly watered to leach nutrients released by the sterilization procedure^55^. After two days of watering, 50 seeds of either *Phleum* or *Poa* were sown into each microcosm. Microcosms with microarthropod addition treatment received three individuals of the predatory mite and ten individuals of each springtail species (30 springtail individuals per microcosm in total, equivalent to 8000 individuals per square meter). The number of added springtails was expected to be sufficient to establish viable population in each microcosm: springtails are known to quickly reach population carrying capacity according to the available resources^56,57^. We did not determine the densities of springtails before setting and after harvesting the experiment, but we expect the original numbers to be close to zero (due to large disturbances during the preparation of the conditioning phase) and the final numbers to be at the carrying capacity of each microcosm^58^.

The microcosms were kept in the climate chamber (for details see above) and were watered daily with 20 ml of distilled water. The establishing seedlings were counted every other day. After four weeks, the aboveground and belowground biomass of all seedlings was harvested, dried at 60 °C, and weighed. Soil samples were taken from each microcosm after removal of plant roots: soil from the whole microcosm was homogenized and sampled for subsequent soil analyses. We also sampled plant roots for estimation of mycorrhizal colonization rates.

### Analyses of soil communities

The soil samples taken after the feedback phase of the experiment were used to analyse the composition of soil microbial communities (5 g of fresh soil per sample, frozen at −20 °C), nematode communities (25 g of fresh soil), and substrate-induced soil respiration (5 g of fresh soil) to determine active soil microbial biomass. We used root samples from the feedback phase to estimate the mycorrhizal root colonisation. In all cases, we used five samples per treatment (i.e., from all replicates), except for only three samples per treatment for analysis of soil microbial communities (PLFAs).

Phospholipid fatty acids (PLFAs) were used as taxonomic markers for the quantification and classification of microorganisms^59^. Analysing PLFAs present in soil samples is an effective tool for studying the composition of soil microbial communities. Before PLFA extraction, soil samples were sieved with 2 mm mesh size to remove root and litter pieces. Lipid extraction followed the protocol by Frostegård *et al*.^60^. Therefore, 5 g fresh weight of soil was weighed and mixed with 18.5 ml Bligh & Dyer reagent^61^. Samples were shaken for 2 h to extract lipids from microorganisms. A centrifugation step with 1500 g for 10 min separated two phases. The upper organic phase was extracted and mixed with 6.2 ml chloroform and 6.2 ml citrate buffer. After centrifugation, 4 ml of the lower phase was transferred to a new tube. This organic phase was evaporated at 30 °C under a nitrogen atmosphere. Silica-gel separation columns fractionated the lipid phase into phospholipid fatty acids by adding methanol which was again evaporated under a nitrogen atmosphere at 30 °C. After evaporation, 30 ml of an internal standard (C 19:0), 1 ml methanol/toluene, and 1 ml methanol/KOH were added. The resulting fatty acid methyl esters (FAME) were heated for 15 min at 37 °C. After adding 2 ml hexanechloroform, 0.3 ml acetic acid, and 2 ml deionized water, the mixture was centrifuged and the upper phase was filled in a new tube and evaporated at 30 °C under a nitrogen atmosphere. The evaporated extract was solved in 100 µl hexane and filled into vials for analysis. FAMEs were identified by chromatographic retention time according to standard mixtures (Bacterial Acid Methyl Esters; methyl ester derivatives of a naturally occurring mix of bacterial fatty acids; Sigma Aldrich, St Louis, USA). Identification and quantification of PLFAs was performed by a gas chromatograph (SHIMADZU GC 17 A) equipped with column DB 225MS (length: 60 m; diameter: 0.25 mm; film thickness: 0.25 mm) and hydrogen as carrier gas.The PLFAs detected in the samples were assigned to the major groups of soil organisms: gram-positive bacteria, gram-negative bacteria, saprotrophic fungi, and arbuscular mycorrhizal fungi^59^ (see Table S1 in Supporting Information).

Nematodes were extracted from 25 g of fresh soil samples (the average soil moisture was 13.3%) using a modified Baermann method^62^. The extraction took 72 hours^63^, which should allow even the slower nematode species to migrate from the soil sample and get trapped in the water. After that, nematodes were preserved in 4% formaldehyde. Nematodes were counted, identified to the family level (or genus, if needed for classification to trophic groups) following Bongers^64^, and classified to trophic groups (bacterial feeders, fungal feeders, plant feeders, omnivores, and predators)^65^. The mean number of nematodes extracted per sample was 139.2 (in the non-sterilized treatments). In each sample, we identified 100 individuals (or all nematodes present in the sample, if the total number did not exceed 100 individuals). We extrapolated the numbers of nematodes in each trophic group to the total nematode abundance in a sample and then expressed the numbers as individuals per gram soil dry weight.

We measured substrate-induced soil microbial respiration of 5 g of fresh soil using an O_2_-microcompensation apparatus^66^. After 24 h of adjusting basal respiration, we added 4 mg D-glucose g^−1^ soil dry weight as aqueous solution and determined the respiratory response to substrate addition. The mean of the lowest three readings within the first 10 h (between the initial peak caused by disturbing the soil and the peak caused by microbial growth) was assessed as the maximum initial respiratory response (MIRR; µl O_2_ g^−1^ soil dry weight). Microbial biomass (µg C g^−1^ soil dry weight) was calculated as 38 × MIRR^67^.

To determine the mycorrhizal root colonization, all fine roots from one microcosm were cut, pooled, and a new random sample was taken as a representative sample^68^ and stored in 50% ethanol until further processing. The samples were then cleared and stained with Trypan Blue. Therefore, root samples were carefully rinsed and cleared using 10% solution of KOH at 90 °C for 60 min. Roots were washed again and acidified with 2% solution of lactic acid at 90 °C for 20 min. Finally, the acidic solution was poured off, and the roots were put into 0.05% solution of Trypan Blue in lactoglycerol and heated at 90 °C for 30 min. After staining, the remaining dye was washed out, and roots were stored in phials filled up with lactoglycerol. The mycorrhizal inoculation rates were determined using the gridline intersect method^68^ with 200 intersects per sample at 100 × magnification. However, due to difficulties with distinguishing between arbuscular mycorrhizal fungi and other endophytic fungi in the roots of *Poa*, the mycorrhizal inoculation rates were determined only in *Phleum* roots.

### Data analysis

We analyzed the data on individual plant measures using ANOVA with the main effects and all possible interactions of the four treatments, i.e., soil conditioning, sterilization, arthropod addition, and sown species. Prior the analyses, we checked the data for normality using QQplot. Further, we checked for correlations of the individual measures and as the total plant biomass was highly correlated with aboveground biomass, we excluded the total biomass from the analyses (see Table S2 in Supporting Information for the correlation matrix). Thus, we performed ANOVA for data on seedling establishment, aboveground and belowground plant biomass, root/shoot ratio, and microbial biomass. If significant differences were found, the means of individual treatments were compared by Tukey’s HSD test. Both data on the total nematode counts and the mycorrhizal colonization of *Phleum* roots were not normally distributed: there were essentially no records of both measures in the sterilized soils, while the values in non-sterilized soils followed a normal distribution. Therefore, we used chi-square tests to analyze the effect of sterilization treatment on the presence of nematodes and the presence of mycorrhiza in *Phleum* roots. Values lower than 2 nematodes per sample (25 g of soil) and values lower than 2 mycorrhizal counts (out of 200 intersects) were treated as zeros. These zeros corresponded to the sterilized treatments. Further, we investigated the effects of soil conditioning, arthropod addition, sown species, and their interactions on nematode counts in non-sterilized soils using ANOVA. We used ANOVA to test the effects of soil conditioning, arthropod addition, and their interaction on mycorrhizal colonization of *Phleum* roots in non-sterilized soils. All statistical analyses were performed using R 2.4.0^69^.

To examine the influence of experimental treatments on the trophic composition of soil nematodes and PLFAs we used redundancy analysis (RDA)^70^. Here, arthropod addition was treated as an explanatory variable and the nematode abundance of each trophic group per gram dry soil (or PLFA measures) as dependent variables, respectively. We repeated this analysis to investigate the effect of sown species, soil conditioning, sterilization, and all possible interactions of these factors. In each analysis, the other main factors were used as covariates (in the case of higher-level interactions we used also all the lower-level interactions as covariates). For visualization of the data of both nematode trophic groups and soil PLFAs, we used principal component analysis (PCA)^70^. PCA is an indirect alternative of RDA searching for the main gradients in the data without explicitly considering the information on the different treatments. We used this method since it reveals the largest possible variance in the data and illustrates the composition of individual samples as well as their similarities. All the multivariate analyses were performed using Canoco 5^71^.

### Data Availability

The data reported in this paper has been deposited at FigShare (10.6084/m9.figshare.6797432).

## Electronic supplementary material

Supporting information
