# Supplementary material for: Soil microarthropods alter the outcome of plant-soil feedback experiments
Source: Sci Rep. 2018 Aug 9;8:11898. doi: 10.1038/s41598-018-30340-w (PMC6085370; doi:10.1038/s41598-018-30340-w)
Supplement: Supplementary file 1 — Supporting information [file 41598_2018_30340_MOESM1_ESM.docx]

**Soil microarthropods alter the outcome of plant-soil feedback experiments**

Eliška Kuťáková^a,b,^*, Simone Cesarz^c,d,e^, Zuzana Münzbergová^a,b^ and Nico Eisenhauer^c,d,e^

* author for correspondence; [kutakova.eliska@gmail.com](mailto:kutakova.eliska@gmail.com)

^a^ Department of Botany, Faculty of Science, Charles University in Prague, Benátská 2, 128 01 Praha 2, Czech Republic

^b^ Institute of Botany, Czech Academy of Sciences, v. v. i., Zámek 1, 252 43 Průhonice, Czech Republic

^c^ German Centre for Integrative Biodiversity Research (iDiv) Halle-Jena-Leipzig, Deutscher Platz 5e, 04103 Leipzig, Germany

^d^ Institute of Biology, Leipzig University, Deutscher Platz 5e, 04103 Leipzig, Germany

^e^Friedrich Schiller University of Jena, Institute of Ecology, Dornburger Str. 159, 07743 Jena, Germany

**Supporting information**

Additional supporting information can be found for this article:

- Table S1: List of PLFAs and assigned groups of soil organisms.
- Table S2: Correlation matrix (Pearson’s correlation coefficient) of all measured plant and soil characteristics.
- Figure S1. Seedling establishment as affected by the interaction of soil conditioning and sterilization treatment.
- Figure S2. Aboveground biomass of *Phleum pratense* and *Poa pratensis* as affected by the interaction of conditioning × sterilization × species.
- Figure S3. Belowground biomass as affected by the interaction of soil conditioning × species.
- Figure S4. Root/shoot ratio as affected by the interaction of soil conditioning × sterilization × species.
- Figure S5. Microbial biomass as affected by the interaction of conditioning × sterilization, and species × sterilization.

**Table S1:** List of PLFAs and assigned groups of organisms.

| **PLFA** | **marker** |
| --- | --- |
| i14:0 | G+ bacteria |
| i15:0 | G+ bacteria |
| a15:0 | G+ bacteria |
| i16:0 | G+ bacteria |
| 16:1ω7 | bacteria widespread |
| i17:0 | G+ bacteria |
| a17:0 | G+ bacteria |
| cy17:0 | G- bacteria |
| i18:0 | G+ bacteria |
| 18:1ω7 | bacteria widespread |
| 18:1ω9 | fungal |
| 18:2ω6,9 | fungal |
| cy19:0 | G- bacteria |
| 20:1ω9 | AM fungi |

| **Table S2:** Correlation matrix (Pearson’s correlation coefficient) for all variables (or PCA scores in case of multivariate soil properties). Positive values mean a positive correlation of the two variables, negative values mean negative correlation. Bold values indicate significant correlations (p<0.05), values in italics and bold indicate marginal significance (p<0.1). | | | | | | | | | | | | | | | | | | | | | | | | | | | | | | | | | | | | | | | | | | | | |
| --- | --- | --- | --- | --- | --- | --- | --- | --- | --- | --- | --- | --- | --- | --- | --- | --- | --- | --- | --- | --- | --- | --- | --- | --- | --- | --- | --- | --- | --- | --- | --- | --- | --- | --- | --- | --- | --- | --- | --- | --- | --- | --- | --- | --- |
|  |  |  | Germination | |  | Biomass | | | | | | | | |  | | | | Root/shoot ratio | |  | Mycorrhiza | | |  | Soil respiration | |  | | Nematode counts | |  | | Nematode PCA scores | | | |  | | PLFAs PCA scores | | | | |
|  |  |  |  |  |  | Aboveground | |  | | Belowground | |  | | Total | | |  | |  |  |  |  |  |  |  |  |  |  | |  |  |  | | 1st axis | | 2nd axis | |  | | 1st axis | | 2nd axis | |  |
| Germination | |  | 1 |  | **0.26** | |  | | **0.44** | |  | | **0.30** | | |  | | **0.23** | |  | 0.04 | |  | **0.30** | | |  | | 0.01 | |  | | 0.03 | | -0.06 | |  | | **0.40** | | **0.49** | |  |  |
| Biomass | Aboveground |  |  | |  | 1 | |  | | **0.74** | |  | | **1.0** | | |  | | **-0.47** | |  | **-0.84** | | |  | **0.50** | |  | | **-0.64** | |  | | **-0.72** | | -0.15 | |  | | **0.75** | | -0.13 | |  |
|  | Belowground |  |  | |  |  | |  | | 1 | |  | | **0.81** | | |  | | ***0.19*** | |  | ***-0.31*** | | |  | **0.24** | |  | | **-0.27** | |  | | **-0.31** | | -0.13 | |  | | **0.53** | | ***0.25*** | |  |
|  | Total |  |  | |  |  | |  | |  | |  | | 1 | | |  | | **-0.39** | |  | **-0.81** | | |  | **0.48** | |  | | **-0.61** | |  | | **-0.70** | | -0.15 | |  | | **0.75** | | -0.08 | |  |
| Root/shoot ratio | |  |  |  |  | |  | |  | |  | |  | | |  | | 1 | |  | **0.72** | |  | **-0.29** | | |  | | **0.62** | |  | | **0.66** | | 0.10 | |  | | **-0.34** | | **0.52** | |  |  |
| Mycorrhiza | |  |  |  |  | |  | |  | |  | |  | | |  | |  | |  | 1 | |  | **-0.63** | | |  | | **0.72** | |  | | **0.88** | | 0.15 | |  | | **-0.68** | | **0.45** | |  |  |
| Soil respiration | |  |  |  |  | |  | |  | |  | |  | | |  | |  | |  |  | |  | 1 | | |  | | **-0.27** | |  | | **-0.31** | | -0.02 | |  | | ***0.28*** | | 0.10 | |  |  |
| Nematode counts | |  |  |  |  | |  | |  | |  | |  | | |  | |  | |  |  | |  |  | | |  | | 1 | |  | | **0.94** | | -0.05 | |  | | **-0.61** | | **0.48** | |  |  |
| Nem. | 1st axis |  |  | |  |  | |  | |  | |  | |  | | |  | |  | |  |  | | |  |  | |  | |  | |  | | 1 | | -0.01 | |  | | **-0.68** | | **0.52** | |  |
|  | 2nd axis |  |  | |  |  | |  | |  | |  | |  | | |  | |  | |  |  | | |  |  | |  | |  | |  | |  | | 1 | |  | | 0.03 | | 0.21 | |  |
|  |  |  |  | |  |  | |  | |  | |  | |  | | |  | |  | |  |  | | |  |  | |  | |  | |  | |  | |  | |  | |  | |  | |  |
| PLFAs | 1st axis |  |  | |  |  | |  | |  | |  | |  | | |  | |  | |  |  | | |  |  | |  | |  | |  | |  | |  | |  | | 1 | | 0.01 | |  |
|  | 2nd axis |  |  | |  |  | |  | |  | |  | |  | | |  | |  | |  |  | | |  |  | |  | |  | |  | |  | |  | |  | |  | | 1 | |  |


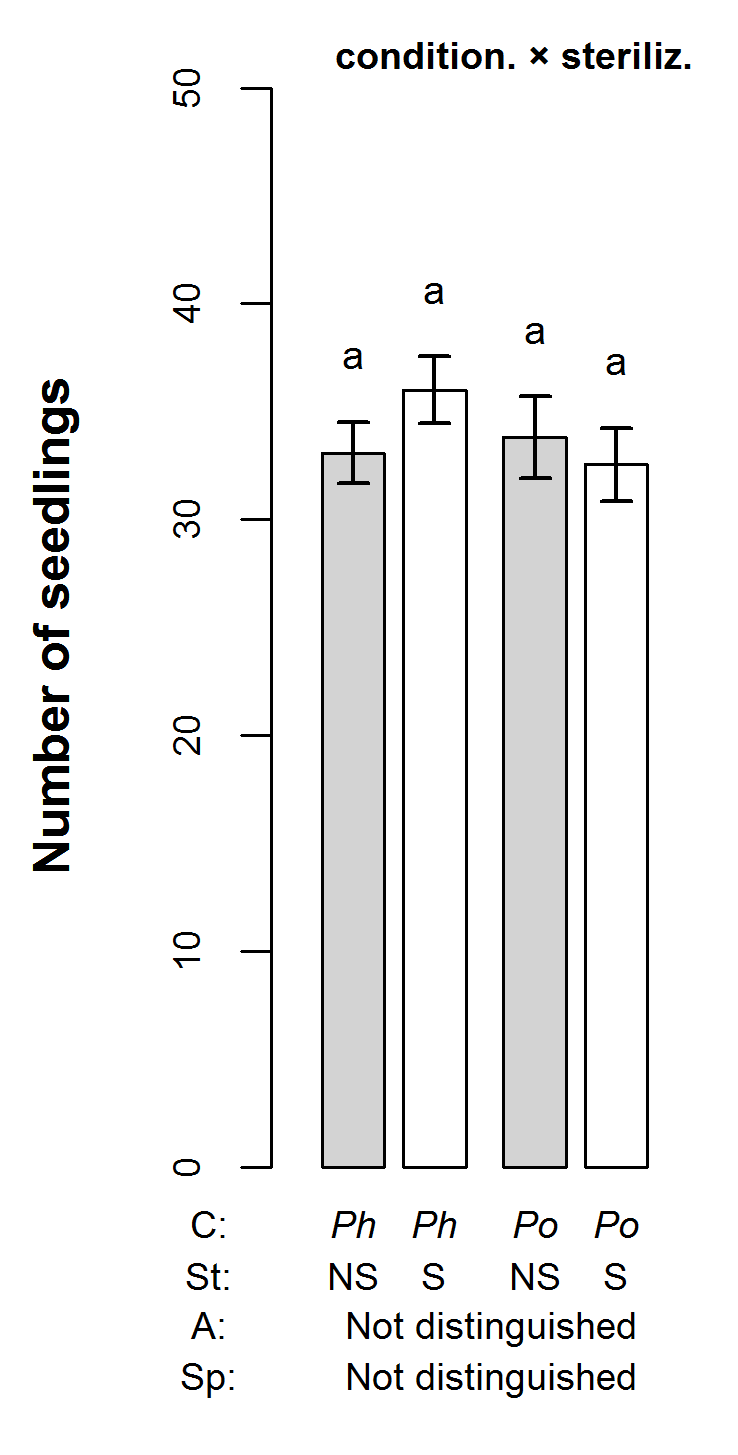


Figure S1: Seedling establishment as affected by the interaction of conditioning × sterilization. Shown are means ± SE. Bars with different letters differ significantly based on Tukey’s HSD test (p<0.05). Grey bars represent non-sterilized soil, white bars sterilized soil. C=conditioning species, Ph=Phleum, Po=Poa; St=sterilization, NS=non-sterilized, S=sterilized; A=arthropod treatment; Sp=plant species in feedback phase; Not distinguished=this treatment is not distinguished in the analysis.


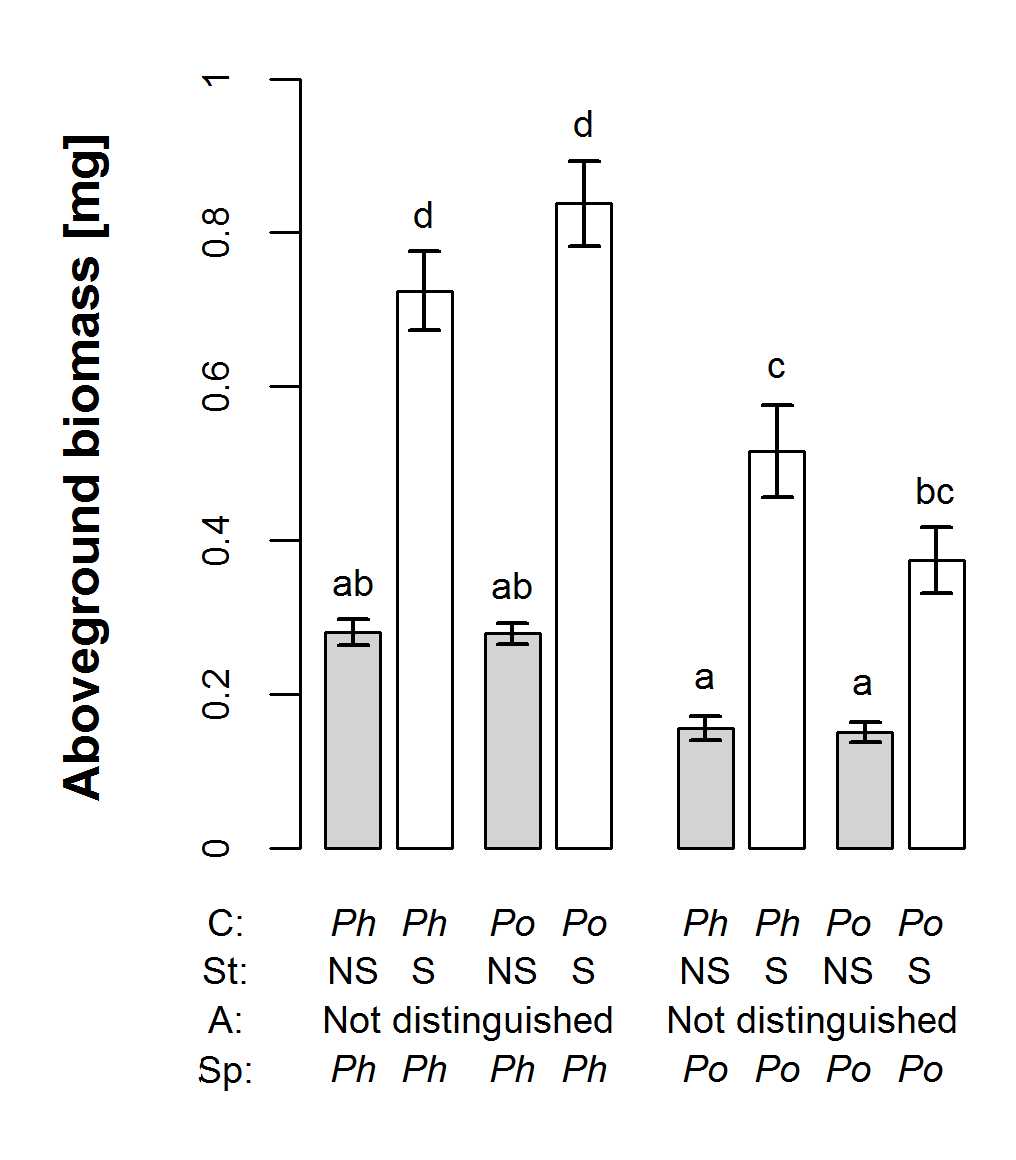


Figure S2. Aboveground biomass of Phleum pratense and Poa pratensis as affected by the interaction of conditioning × sterilization × species. Shown are means ± SE. Bars with different letters differ significantly based on Tukey’s HSD test (p<0.05). Grey bars represent non-sterilized soil, white bars sterilized soil. C=conditioning species, Ph=Phleum, Po=Poa; St=sterilization, NS=non-sterilized, S=sterilized; A=arthropod treatment; Sp=plant species in feedback phase; Not distinguished=this treatment is not distinguished in the analysis.


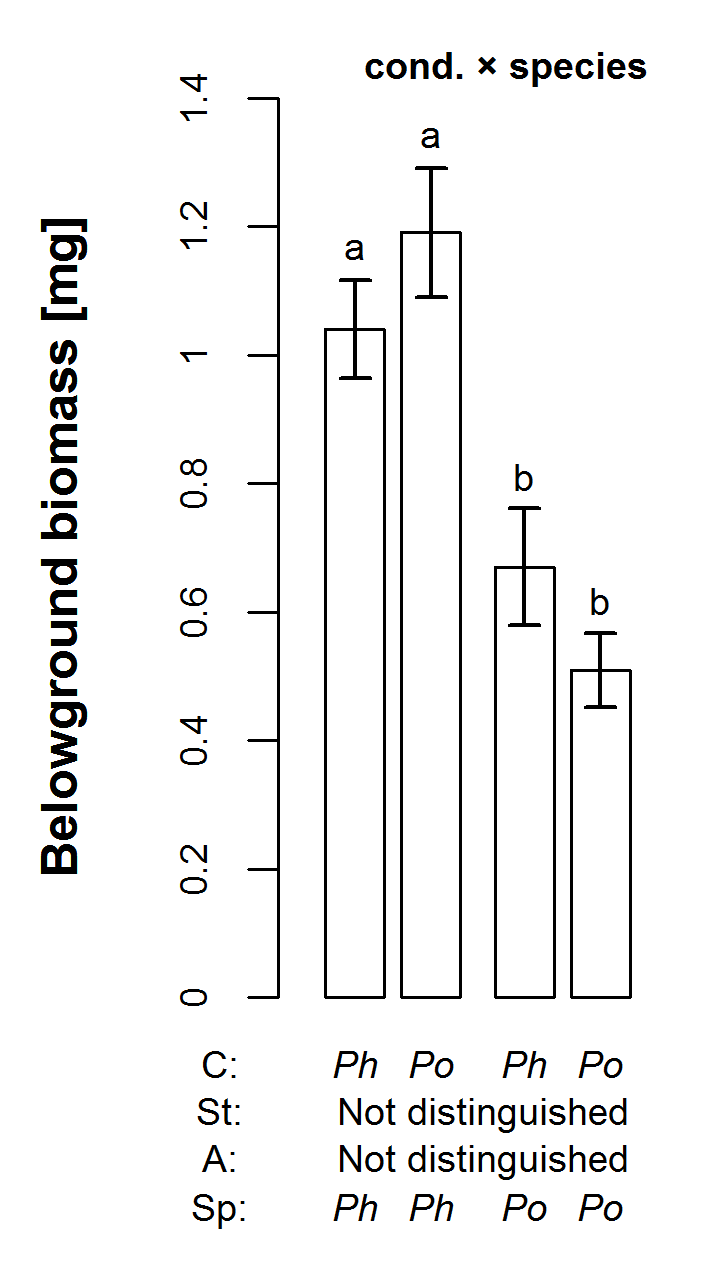


Figure S3. Belowground biomass as affected by the interaction of soil conditioning × species. Shown are means ± SE. Bars with different letters differ significantly based on Tukey’s HSD test (p<0.05). C=conditioning species, Ph=Phleum, Po=Poa; St=sterilization, NS=non-sterilized, S=sterilized; A=arthropod treatment; Sp=plant species in feedback phase; Not distinguished=this treatment is not distinguished in the analysis.


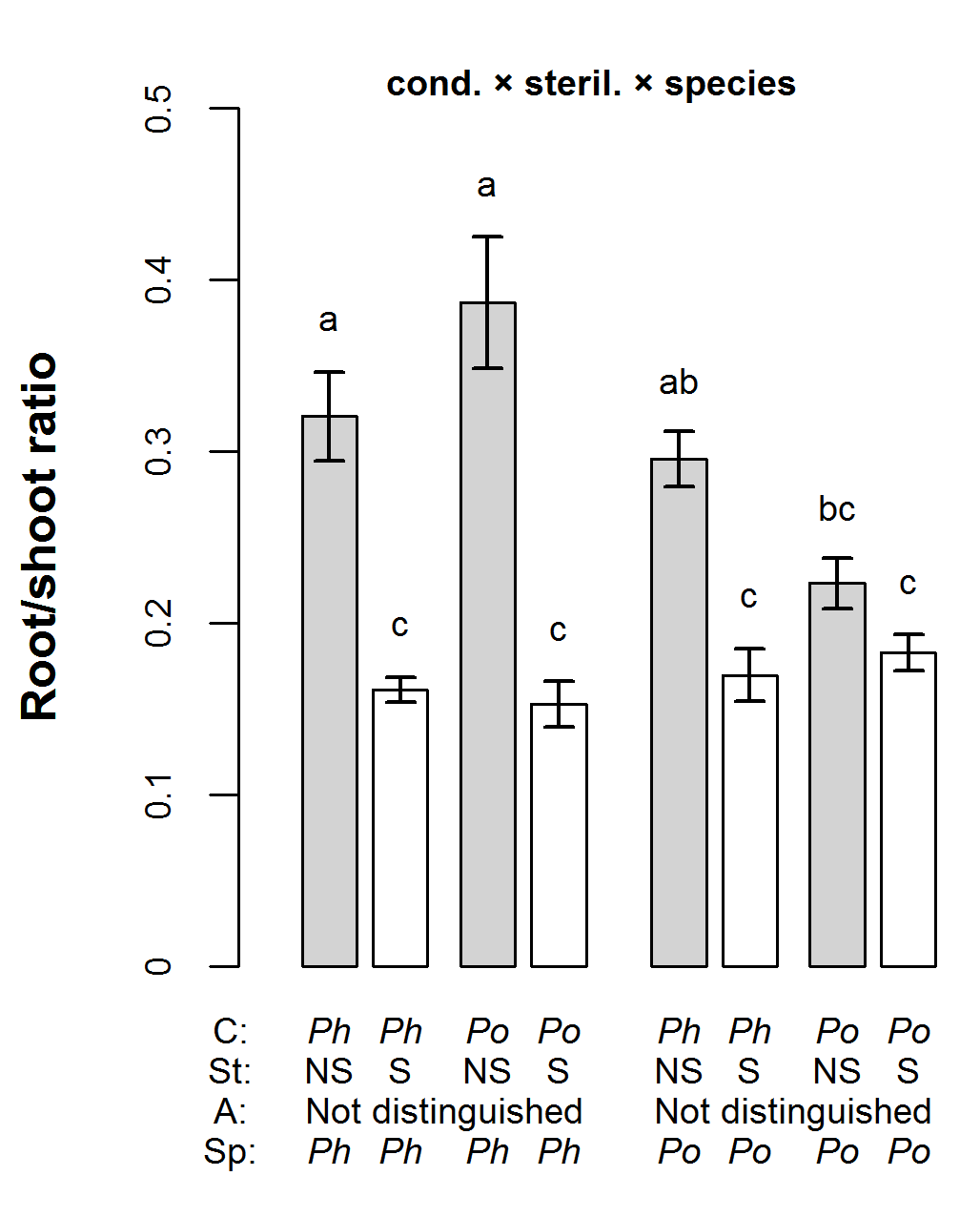


Figure S4. Root-to-shoot ratio as affected by the interaction of soil conditioning × sterilization × species. Shown are means ± SE. Bars with different letters differ significantly based on Tukey’s HSD test (p<0.05). Grey bars represent non-sterilized soil, white bars sterilized soil. C=conditioning species, Ph=Phleum, Po=Poa; St=sterilization, NS=non-sterilized, S=sterilized; A=arthropod treatment; Sp=plant species in feedback phase; Not distinguished=this treatment is not distinguished in the analysis.


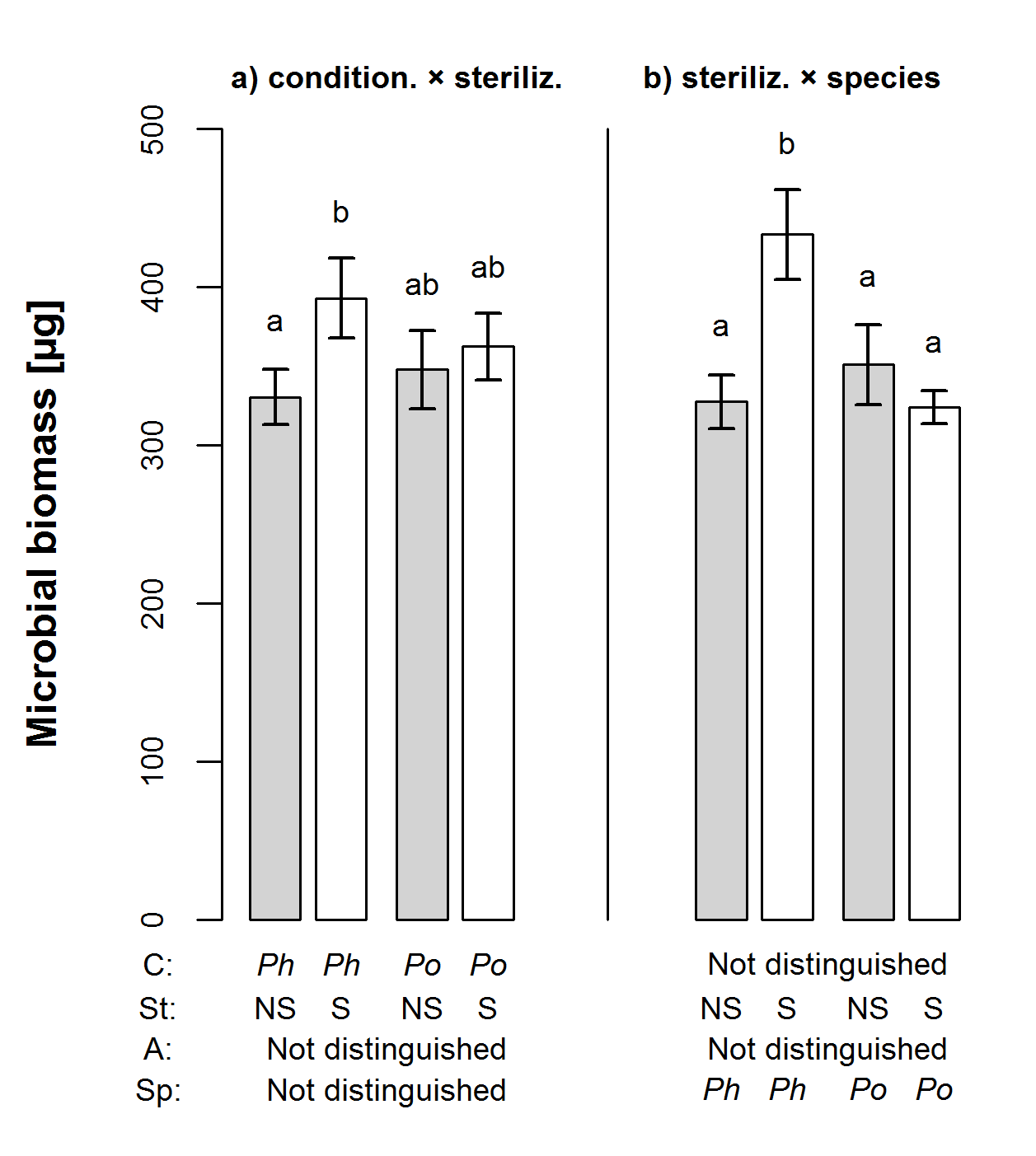


Figure S5. Microbial biomass as influenced by the interaction of a) conditioning and sterilization, and b) species and sterilization. Shown are means ± SE. Bars with different letters differ significantly based on Tukey’s HSD test (p<0.05). Grey bars represent non-sterilized soil, white bars sterilized soil. C=conditioning species, Ph=Phleum, Po=Poa; St=sterilization, NS=non-sterilized, S=sterilized; A=arthropods; Sp=plant species in feedback phase; Not distinguished=this treatment is not distinguished in the respective analysis.
